# A biased activation theory of the cognitive and attentional modulation of emotion

**DOI:** 10.3389/fnhum.2013.00074

**Published:** 2013-03-18

**Authors:** Edmund T. Rolls

**Affiliations:** Oxford Centre for Computational NeuroscienceOxford, UK

**Keywords:** cognition, emotion, orbitofrontal cortex, decision-making, the noisy brain, planning

## Abstract

Cognition can influence emotion by biasing neural activity in the first cortical region in which the reward value and subjective pleasantness of stimuli is made explicit in the representation, the orbitofrontal cortex (OFC). The same effect occurs in a second cortical tier for emotion, the anterior cingulate cortex (ACC). Similar effects are found for selective attention, to for example the pleasantness vs. the intensity of stimuli, which modulates representations of reward value and affect in the orbitofrontal and anterior cingulate cortices. The mechanisms for the effects of cognition and attention on emotion are top-down biased competition and top-down biased activation. Affective and mood states can in turn influence memory and perception, by backprojected biasing influences. Emotion-related decision systems operate to choose between gene-specified rewards such as taste, touch, and beauty. Reasoning processes capable of planning ahead with multiple steps held in working memory in the explicit system can allow the gene-specified rewards not to be selected, or to be deferred. The stochastic, noisy, dynamics of decision-making systems in the brain may influence whether decisions are made by the selfish-gene-specified reward emotion system, or by the cognitive reasoning system that explicitly calculates reward values that are in the interests of the individual, the phenotype.

## Introduction

How do cognition and attention influence brain processing of emotion-provoking, that is affective, stimuli? What are the neural mechanisms?

To address this I review some of the experimental evidence on how cognition and selective attention influence the neural processing of affective stimuli.

Then I describe a top-down biased activation theory of emotion that provides a mechanism by which cognition and attention influence emotion and emotion-provoking stimuli.

The emphasis of the paper is on providing a fundamental framework at the level of brain computation for understanding how cognition and emotion influence each other, and how decisions are made between an emotional system that has its origins in gene-specified rewards, and an explicit reasoning system that allows these rewards to be deferred in favor of long-term reward value in the interests of the individual (Rolls, 2014). The approach is based on research by the author and his colleagues, and complementary research is cited below.

First, I outline an approach (Rolls, 2013b, 2014) to what emotions are, and what stimuli elicit emotions, to provide a clear foundation for what processes the cognitive and attentional inputs must influence.

### A definition of emotional states

Emotions can usefully be defined (operationally) as states elicited by rewards and punishers which have particular functions (Rolls, 1999, 2005, 2013b, 2014). The functions are defined below, and include working to obtain or avoid the rewards and punishers. A reward is anything for which an animal (which includes humans) will work. A punisher is anything that an animal will escape from or avoid. An example of an emotion might thus be the happiness produced by being given a particular reward, such as a pleasant touch, praise, or winning a large sum of money. Another example of an emotion might be fear produced by the sound of a rapidly approaching bus, or the sight of an angry expression on someone's face. We will work to avoid such stimuli, which are punishing. Another example would be frustration, anger, or sadness produced by the omission of an expected reward, or the termination of a reward such as the death of a loved one. Another example would be relief, produced by the omission or termination of a punishing stimulus such as the removal of a painful stimulus, or sailing out of danger. These examples indicate how emotions can be produced by the delivery, omission, or termination of rewarding or punishing stimuli, and go some way to indicate how different emotions could be produced and classified in terms of the rewards and punishers received, omitted, or terminated.

I consider elsewhere a slightly more formal definition than rewards or punishers, in which the concept of reinforcers is introduced, and it is shown that emotions can be usefully seen as states produced by instrumental reinforcing stimuli (Rolls, 2005, 2014). Instrumental reinforcers are stimuli which, if their occurrence, termination, or omission is made contingent upon the making of a response, alter the probability of the future emission of that response. Some stimuli are unlearned reinforcers (e.g., the taste of food if the animal is hungry, or pain); while others may become reinforcing by associative learning, because of their association with such primary reinforcers, thereby becoming “secondary reinforcers.”

**Figure 1:**
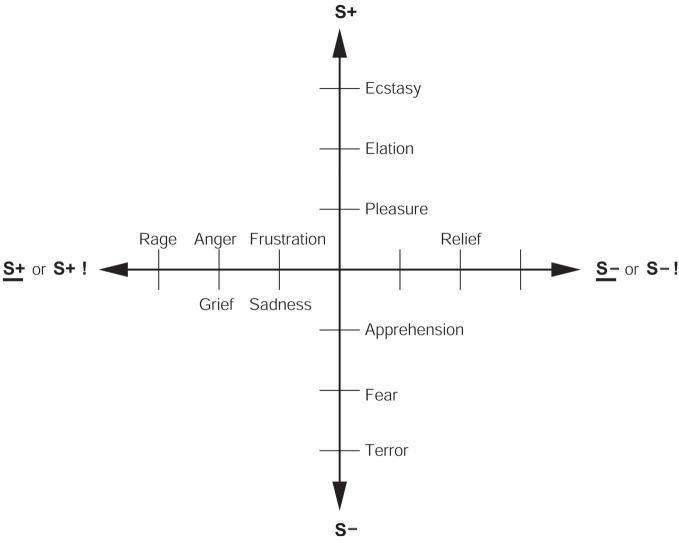
**Some of the emotions associated with different reinforcement contingencies are indicated.** Intensity increases away from the center of the diagram, on a continuous scale. The classification scheme created by the different reinforcement contingencies consists of (1) the presentation of a positive reinforcer (S+), (2) the presentation of a negative reinforcer (S−), (3) the omission of a positive reinforcer (S+) or the termination of a positive reinforcer (S+ !), and (4) the omission of a negative reinforcer (S−) or the termination of a negative reinforcer (S− !). It should be understood that each different reinforcer will produce different emotional states: this diagram just summarizes the types of emotion that may be elicited by different contingencies, but the actual emotions will be different for each reinforcer (see Rolls, 2014).

This foundation has been developed (Rolls, 2005) to show how a very wide range of emotions can be accounted for, as a result of the operation of a number of factors, including the following:

1. The *reinforcement contingency* (e.g., whether reward or punishment is given, or withheld) (see Figure 1).
2. The *intensity* of the reinforcer (see Figure 1).
3. Any environmental stimulus might have a *number of different reinforcement associations*. (For example, a stimulus might be associated both with the presentation of a reward and of a punisher, allowing states such as conflict and guilt to arise).
4. Emotions elicited by stimuli associated with *different primary reinforcers* will be different.
5. Emotions elicited by *different secondary reinforcing stimuli* will be different from each other (even if the primary reinforcer is similar).
6. The emotion elicited can depend on whether an *active or passive behavioral response* is possible. (For example, if an active behavioral response can occur to the omission of a positive reinforcer, then anger might be produced, but if only passive behavior is possible, then sadness, depression, or grief might occur).

By combining these six factors, it is possible to account for a very wide range of emotions (Rolls, 2005, 2014).

### The functions of emotion

The functions of emotion also provide insight into the nature of emotion. These functions, described more fully elsewhere (Rolls, 2005), can be summarized as follows:

1. The *elicitation of autonomic responses* (e.g., a change in heart rate) and *endocrine responses* (e.g., the release of adrenaline). These prepare the body for action.
2. *Flexibility of behavioral responses to reinforcing stimuli*. Emotional (and motivational) states allow a simple interface between sensory inputs and action systems. The essence of this idea is that goals for behavior are specified by reward and punishment evaluation. When an environmental stimulus has been decoded as a primary reward or punishment, or (after previous stimulus-reinforcer association learning) a secondary rewarding or punishing stimulus, then it becomes a goal for action. The human can then perform any action (instrumental action) to obtain the reward, or to avoid the punisher. Thus there is flexibility of action, and this is in contrast with stimulus-response, or habit, learning in which a particular response to a particular stimulus is learned. The emotional route to action is flexible not only because any action can be performed to obtain the reward or avoid the punishment, but also because the human can learn in as little as one trial that a reward or punishment is associated with a particular stimulus, in what is termed “stimulus-reinforcer association learning.”

Selecting between available rewards with their associated costs, and avoiding punishers with their associated costs, is a process that can take place both implicitly (unconsciously), and explicitly using a language system to enable long-term plans to be made (Rolls, 2005, 2008b). These many different brain systems, some involving implicit evaluation of rewards, and others explicit, verbal, conscious, evaluation of rewards and planned long-term goals, must all enter into the selector of behavior.

The implication is that operation by animals (including humans) using reward and punishment systems tuned to dimensions of the environment that increase fitness provides a mode of operation that can work in organisms that evolve by natural selection. It is clearly a natural outcome of Darwinian evolution to operate using reward and punishment systems tuned to fitness-related dimensions of the environment, if arbitrary responses are to be made by the animals, rather than just preprogrammed movements such as tropisms, taxes, and reflexes. This view of brain design in terms of reward and punishment systems built by genes that gain their adaptive value by being tuned to a goal for action offers I believe a deep insight into how natural selection has shaped many brain systems, and is a fascinating outcome of Darwinian thought (Rolls, 2005, 2011b, 2014).

**Figure 2:**
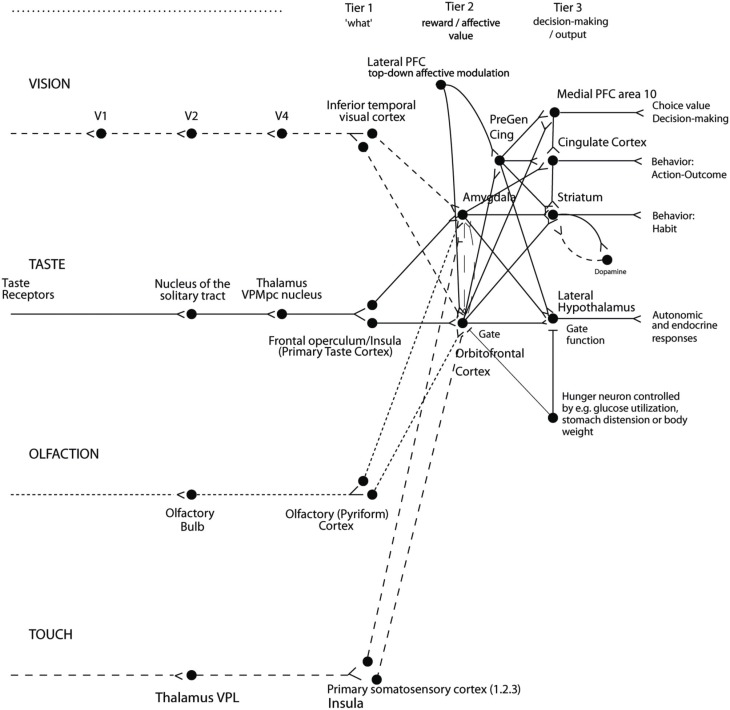
**Organization of cortical processing for computing value (in Tier 2) and making value-based decisions (in Tier 3) and interfacing to action systems.** The Tier 1 brain regions up to and including the column headed by the inferior temporal visual cortex compute and represent neuronally “what” stimulus/object is present, but not its reward or affective value. Tier 2 represents by its neuronal firing the reward or affective value, and includes the orbitofrontal cortex, amygdala, and anterior including pregenual cingulate cortex. Tier 3 is involved in choices based on reward value (in particular VMPFC area 10), and in different types of output to behavior. The secondary taste cortex, and the secondary olfactory cortex, are within the orbitofrontal cortex. V1—primary visual cortex. V4—visual cortical area V4. PreGen Cing—pregenual cingulate cortex. “Gate” refers to the finding that inputs such as the taste, smell, and sight of food in regions where reward value is represented only produce effects when an appetite for the stimulus (modulated for example by hunger) is present (Rolls, 2005). Lateral PFC: lateral prefrontal cortex, a source for top-down attentional and cognitive modulation of affective value (Grabenhorst and Rolls, 2010). This is a schematic diagram, and is based on primates including humans, as rodents appear not to have homologs of some of the areas shown, including the granular prefrontal cortex, which includes much of the orbitofrontal cortex (Wise, 2008; Passingham and Wise, 2012); and because rodents have a taste system that is connected differently, without the obligatory route to the cortex that is shown (Scott and Small, 2009; Rolls, 2013a, 2014).

The implication in the current context is that we are interested in processing in brain systems where instrumental rewards and punishers, and how the processing in these brain systems is modulated by cognition and by selective attention. A large amount of evidence shows that reward processing occurs in a tier of structures involving the orbitofrontal cortex (OFC) and amygdala (see Figure 2) (Rolls, 2014). At the preceding stages of processing, the representations are not of reward value, but instead of what taste is present and its intensity (the primary taste cortex), what odor is present (the pyriform cortex), and what visual stimulus is present (the inferior temporal visual cortex) (see Figure 2) (Rolls, 2014).

## Effects of cognition on emotion

To what extent does cognition influence the hedonics of stimuli that produce emotions, and how far down into the sensory system does the cognitive influence reach? Examples of the evidence on this are considered next. Further examples of the effects of cognition on emotion are described elsewhere (Grabenhorst and Rolls, 2011; Shackman et al., 2011; Lindquist et al., 2012; Ochsner et al., 2012; Sheppes et al., 2012; Rolls, 2014).

### Effects of cognition on olfactory and taste reward-related processing

To address this, we performed an fMRI investigation in which the delivery of a standard test odor (isovaleric acid combined with cheddar cheese odor, presented orthonasally using an olfactometer) was paired with a descriptor word on a screen, which on different trials was “cheddar cheese” or “body odor.” Participants rated the affective value of the test odor as significantly more pleasant when labeled “cheddar cheese” than when labeled “body odor,” and these effects reflected activations in the medial OFC/rostral anterior cingulate cortex (ACC) that had correlations with the pleasantness ratings (de Araujo et al., 2005). The implication is that cognitive factors can have profound effects on our responses to the hedonic properties of affective stimuli, in that these effects are manifest quite far down into sensory processing, in that hedonic representations of odors are affected (de Araujo et al., 2005).

**Figure 3:**
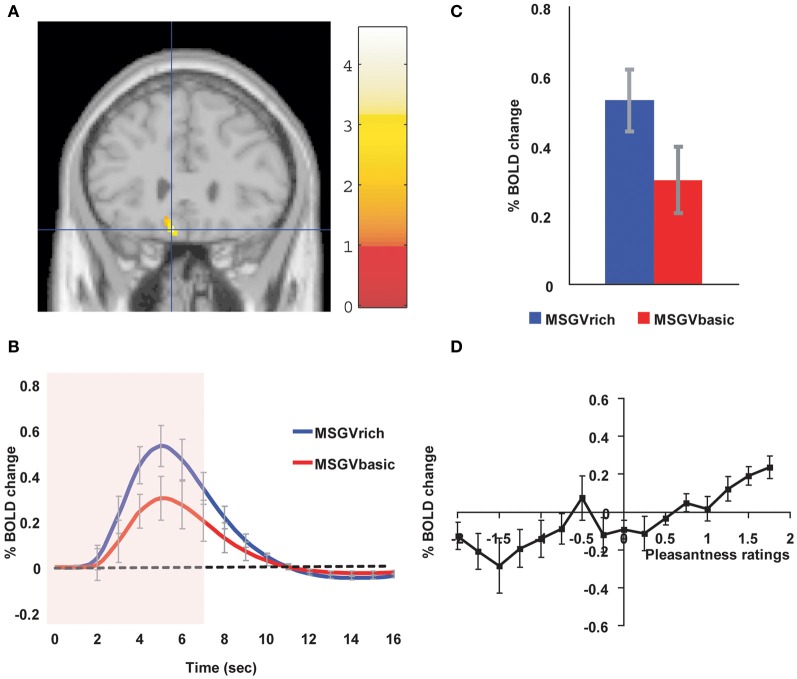
**Cognitive modulation of flavor reward processing in the brain. (A)** The medial orbitofrontal cortex was more strongly activated when a flavor stimulus was labeled “rich and delicious flavor” (MSGVrich) than when it was labeled “boiled vegetable water” (MSGVbasic) ([−8 28 −20]). (The flavor stimulus, MSGV, was the taste 0.1 M MSG + 0.005 M inosine 5′monophosphate combined with a consonant 0.4% vegetable odor). **(B)** The timecourse of the BOLD signals for the two conditions. **(C)** The peak values of the BOLD signal (mean across subjects ± SEM) were significantly different (*t* = 3.06, *df* = 11, *p* = 0.01). **(D)** The BOLD signal in the medial orbitofrontal cortex was correlated with the subjective pleasantness ratings of taste and flavor, as shown by the SPM analysis, and as illustrated (mean across subjects ± SEM, *r* = 0.86, *p* < 0.001). [Reproduced with permission from Grabenhorst et al. (2008)].

Similar cognitive effects and mechanisms have now been found for the taste and flavor of food, where the cognitive word level descriptor was for example “rich delicious flavor” and activations to flavor were increased in the OFC and regions to which it projects including the pregenual cingulate cortex and ventral striatum, but were not influenced in the insular primary taste cortex where activations reflected the intensity (concentration) of the stimuli (Grabenhorst et al., 2008) (see Figure 3).

### Effects of cognition on touch reward-related processing

#### The representation of positively affective touch and temperature in the brain

While there have been many investigations of the neural representations of pain stimuli (Grabenhorst and Rolls, 2011; Shackman et al., 2011; Kobayashi, 2012), there have been fewer investigations of the representation of pleasant touch in the brain.

In one study, the cortical areas that represent affectively positive and negative aspects of touch were investigated using functional magnetic resonance imaging (fMRI) by comparing activations produced by pleasant touch, painful touch produced by a stylus, and neutral touch, to the left hand (Rolls et al., 2003c). It was found that regions of the OFC were activated more by pleasant touch and by painful stimuli than by neutral touch, and that different areas of the OFC were activated by the pleasant and painful touches. The OFC activation was related to the affective aspects of the touch, in that the somatosensory cortex (S1) was less activated by the pleasant and painful stimuli than by the neutral stimuli (as shown by a Two-Way analysis of variance performed on the percentage change of the BOLD signals under the different stimulation conditions in the different areas). Further, it was found that a rostral part of the ACC was activated by the pleasant stimulus and that a more posterior and dorsal part was activated by the painful stimulus [and this is consistent with effects in other sensory modalities (Grabenhorst and Rolls, 2011; Rolls, 2014) (cf. Etkin et al., 2011)]. Regions of the somatosensory cortex, including S1, and part of S2 in the superior temporal plane at the mid-insula level, were activated more by the neutral touch than by the pleasant and painful stimuli. Part of the posterior insula was activated only in the pain condition, and different parts of the brainstem, including the central gray, were activated in the pain, pleasant and neutral touch conditions. The results provide evidence that different areas of the human OFC are involved in representing both pleasant touch and pain, and that dissociable parts of the cingulate cortex are involved in representing pleasant touch and pain (Rolls et al., 2003c).

Warm and cold stimuli have affective components such as feeling pleasant or unpleasant, and these components may have survival value, for approach to warmth and avoidance of cold may be reinforcers or goals for action built into us during evolution to direct our behavior to stimuli that are appropriate for survival (Rolls, 2005). Understanding the brain processing that underlies these prototypical reinforcers provides a direct approach to understanding the brain mechanisms of emotion. In an fMRI investigation in humans, we showed that the mid-orbitofrontal and pregenual cingulate cortex and the ventral striatum have activations that are correlated with the subjective pleasantness ratings made to warm (41°C) and cold (12°C) stimuli, and combinations of warm and cold stimuli, applied to the hand (Rolls et al., 2008b). Activations in the lateral and some more anterior parts of the OFC were correlated with the unpleasantness of the stimuli. In contrast, activations in the somatosensory cortex and ventral posterior insula were correlated with the intensity but not the pleasantness of the thermal stimuli (Rolls et al., 2008b).

A principle thus appears to be that processing related to the affective value and associated subjective emotional experience of somatosensory and thermal stimuli that are important for survival is performed in different brain areas to those where activations are related to sensory properties of the stimuli such as their intensity. This conclusion appears to be the case for processing in a number of sensory modalities, and the finding with such prototypical stimuli as pleasant and painful touch, and warm (pleasant) and cold (unpleasant) thermal stimuli, provides strong support for this principle (Rolls, 2005; Grabenhorst and Rolls, 2008, 2011; Grabenhorst et al., 2008; Rolls et al., 2008a). An implication of the principle is that by having a system specialized for the affective or reward aspects of stimuli it is possible to modify goal oriented behavior, and to do this independently of being able to know what the stimulus is (its intensity, physical characteristics etc). Thus even if a stimulus has lost its pleasantness because of for example a change of core body temperature, it is still possible to represent the stimulus, recognize it, and learn about where it is in the environment for future use (Rolls, 2005). This is a fundamental aspect of brain design (Rolls, 2005, 2008b, 2014).

#### Cognitive modulation of affective touch processing

There have been many studies of the top-down attentional modulation (Rolls, 2008b) of touch, with effects typically larger in secondary somatosensory and association cortical areas (e.g., parietal area 7), and smaller in S1 (Johansen-Berg and Lloyd, 2000; Rolls, 2010). However, there has been little investigation of where high-level cognition influences the representation of affective touch in the brain.

To investigate where cognitive influences from the very high level of language might influence the affective representation of touch, we performed a fMRI study in which the forearm was rubbed with a cream, but this could be accompanied by a word label that indicated that it was a rich moisturizing cream (pleasant to most people) vs. a basic cream (McCabe et al., 2008).

We found that cognitive modulation by a label at the word level indicating pleasantness/richness (“rich moisturizing cream” vs. “basic cream”) influenced the representation of tactile inputs in the OFC (McCabe et al., 2008). (The cream was identical in all conditions in the study: it was only the word labels that were changed. The cream was rubbed onto the ventral surface of the forearm.) For example, a negative correlation with the pleasantness ratings of the touch as influenced by the word labels was found in the lateral OFC, a region shown in other studies to be activated by less pleasant stimuli including unpleasant odors, and losing money (O'Doherty et al., 2001; Rolls et al., 2003b,c). A positive correlation with the pleasantness of touch as influenced by the word labels was found in the pregenual cingulate cortex (McCabe et al., 2008). Convergent evidence on the functions of this region is that the pregenual cingulate region is close to where in different studies another somatosensory stimulus, oral texture, is represented (de Araujo and Rolls, 2004), correlations with pleasantness ratings are found to food and olfactory stimuli (Kringelbach et al., 2003; de Araujo et al., 2005; Grabenhorst and Rolls, 2011), and pleasant touch produces activation (Rolls et al., 2003c). We also found that activations to touch in the parietal cortex area 7 were influenced by the word labels, in that there was more activation when the rich label than when the thin label was present (McCabe et al., 2008).

#### Cognitive modulation of activations to the sight of touch

Cognitive modulation of effects produced by the sight of touch were investigated by a comparison of the effects of the sight of the arm being rubbed when accompanied by the label “rich moisturizing cream” vs. “basic cream.” Cognitive modulation effects were found in the pregenual cingulate cortex extending into the OFC, in regions close to those where activations were correlated with the pleasantness ratings with the same two stimulus conditions. The effect of the cognitive label “rich moisturizing cream” was to make the sight of the touch more pleasant by increasing activations in these pregenual cingulate and OFC areas (McCabe et al., 2008).

## Top-down effects of selective attention on emotion

In section “Effects of Cognition on Emotion”, the effects of cognition on emotion were considered, and cognition referred to for example language-level descriptions of the properties of a stimulus, such as delicious, or rich and moisturizing. In addition, paying selective attention to one property of a stimulus, such as its intensity, vs. another property, such as its pleasantness, can be thought of as a top-down attentional effect. The mechanisms though may be similar, as considered in section “A Top-Down Biased Activation Theory of Attentional and Cognitive Modulation.”

### Taste, olfaction, and flavor

**Figure 4:**
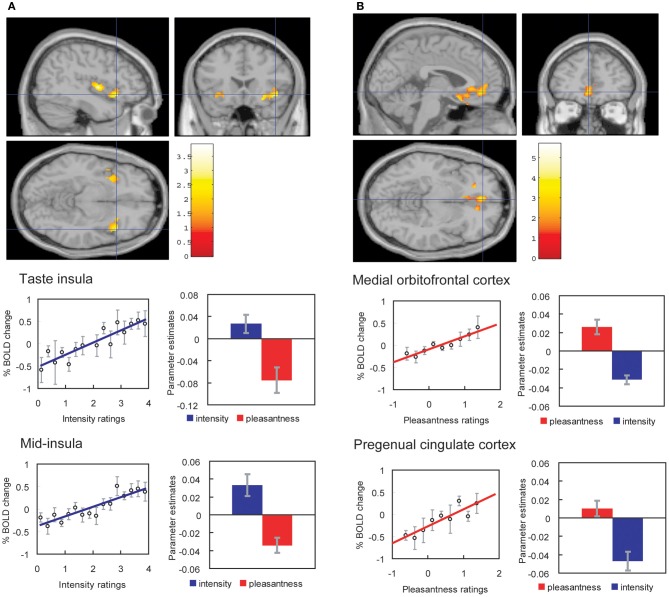
**Effect of paying attention to the pleasantness vs. the intensity of a taste stimulus. (A)** Top: A significant difference related to the taste period was found in the taste insula at [42 18 −14], *z* = 2.42, *p* < 0.05 (indicated by the cursor) and in the mid insula at [40 −2 4], *z* = 3.03, *p* < 0.025. Middle: Taste insula. Right: The parameter estimates (mean ± SEM across subjects) for the activation at the specified coordinate for the conditions of paying attention to pleasantness or to intensity. The parameter estimates were significantly different for the taste insula *t* = 4.5, *df* = 10, *p* = 0.001. Left: The correlation between the intensity ratings and the activation (% BOLD change) at the specified coordinate (*r* = 0.91, *df* = 14, *p* << 0.001). Bottom: Mid insula. Right: The parameter estimates (mean ± SEM across subjects) for the activation at the specified coordinate for the conditions of paying attention to pleasantness or to intensity. The parameter estimates were significantly different for the mid insula *t* = 5.02, *df* = 10, *p* = 0.001. Left: The correlation between the intensity ratings and the activation (% BOLD change) at the specified coordinate (*r* = 0.89, *df* = 15, *p* << 0.001). The taste stimulus, monosodium glutamate, was identical on all trials. **(B)** Top: A significant difference related to the taste period was found in the medial orbitofrontal cortex at [−6 14 −20], *z* = 3.81, *p* < 0.003 (toward the back of the area of activation shown) and in the pregenual cingulate cortex at [−4 46 −8], *z* = 2.90, *p* < 0.04 (at the cursor). Middle: Medial orbitofrontal cortex. Right: The parameter estimates (mean ± SEM across subjects) for the activation at the specified coordinate for the conditions of paying attention to pleasantness or to intensity. The parameter estimates were significantly different for the orbitofrontal cortex *t* = 7.27, *df* = 11, *p* < 10^−4^. Left: The correlation between the pleasantness ratings and the activation (% BOLD change) at the specified coordinate (*r* = 0.94, *df* = 8, *p* << 0.001). Bottom: Pregenual cingulate cortex. Conventions as above. Right: The parameter estimates were significantly different for the pregenual cingulate cortex *t* = 8.70, *df* = 11, *p* < 10^−5^. Left: The correlation between the pleasantness ratings and the activation (% BOLD change) at the specified coordinate (*r* = 0.89, *df* = 8, *p* = 0.001). The taste stimulus, 0.1 M monosodium glutamate, was identical on all trials. [Reproduced with permission from Grabenhorst and Rolls (2008)].

We have found that with taste and flavor (Grabenhorst and Rolls, 2008) stimuli, and olfactory (Rolls et al., 2008a) stimuli, selective attention to pleasantness modulates representations in the OFC (see Figure 4), whereas selective attention to intensity modulates activations in areas such as the primary taste cortex. Thus, depending on the context in which tastes and odors are presented and whether affect is relevant, the brain responds to taste, flavor, and odor, differently.

These findings show that when attention is paid to affective value, the brain systems engaged to represent the stimulus are different from those engaged when attention is directed to the physical properties of a stimulus such as its intensity.

This differential biasing by prefrontal cortex attentional mechanisms (Grabenhorst and Rolls, 2010; Ge et al., 2012) of brain regions engaged in processing a sensory stimulus depending on whether the cognitive demand is for affect-related vs. more sensory-related processing may be an important aspect of cognition and attention which have implications for how strongly the reward system is driven by stimuli including food, and thus for eating and the control of appetite (Grabenhorst and Rolls, 2008, 2011; Rolls et al., 2008a; Rolls, 2012). This important concept is addressed further below.

### Possible sources of the top-down modulation of emotional processing

There is relatively little prior evidence on the top-down source of the bias when attention is to affective (emotional) vs. sensory aspects (e.g., the intensity) of the same stimulus (Pessoa, 2009). In a study using psychophysiological interaction (PPI) analysis, we found that two sites where selective attention to pleasantness increased the activation to taste, the OFC and a region to which it is connected, the pregenual cingulate cortex, both had functional connectivity with a quite anterior (mean *y* ≈ 50) part of the lateral prefrontal cortex, illustrated in Grabenhorst and Rolls (2010). These parts of the OFC and pregenual cingulate cortex are a functionally appropriate target site for a top-down attentional modulation, in that their activations are correlated with the subjectively rated pleasantness of the taste (Grabenhorst and Rolls, 2008). Moreover, the lateral prefrontal cortex has been shown to represent current task sets and attentional demands for different types of tasks (Sakai and Passingham, 2003, 2006).

The statistics used in the calculation of PPI effects (Friston et al., 1997) do not reveal the directionality of the connectivity, for they are based on correlations. However, the directionality in this case is likely to be from the prefrontal cortex to the orbitofrontal and pregenual cingulate cortices, for the following reasons. First, the prefrontal cortex has a powerfully developed recurrent collateral system which provides the basis for the short-term memory (Rolls and Deco, 2002; Deco and Rolls, 2005a; Rolls, 2008b) that is needed to hold the subject of attention active, providing the source of the bias for top-down biased competition (Desimone and Duncan, 1995; Rolls and Deco, 2002; Deco and Rolls, 2005a; Rolls, 2008b). Second, prefrontal cortex lesions impair attention (Beck and Kastner, 2009; Rossi et al., 2009). Third, activations in areas of the lateral prefrontal cortex are related to task set, attentional instructions, and remembering rules that guide task performance (Sakai and Passingham, 2003; Deco and Rolls, 2005a; Sakai and Passingham, 2006; Veldhuizen et al., 2007; Beck and Kastner, 2009; Bengtsson et al., 2009; Kouneiher et al., 2009; Rossi et al., 2009). Fourth, direct anatomical connections exist between the lateral prefrontal cortex and the orbitofrontal and pregenual cingulate cortices (Price, 2006).

The conclusion that these findings suggest is therefore that a part of the lateral prefrontal cortex, not a site normally implicated in affective value and emotion, may be able to modulate emotion-/affect-related processing in the brain by a top-down attentional influence. This may be one way in which higher cognitive functions, such as a reasoning-based strategy and route to action, or verbal instruction to direct processing toward or away from emotion-related brain processing, or conscious volition, can influence the degree to which the affect-related parts of the brain process incoming (or potentially remembered) stimuli that can produce emotional responses. This is thus a part of the way in which cognition can influence, and control, emotion (Rolls, 2005, 2011a, 2014; Pessoa, 2009).

We also found that two sites where selective attention to intensity increased the activation to the taste delivery into the mouth, the anterior and mid insula, both had functional connectivity with a less anterior (mean *y* ≈ 37) part of the lateral prefrontal cortex (Grabenhorst and Rolls, 2010). These parts of the insula are a functionally appropriate site for a top-down attentional modulation, in that their activations are correlated with the subjectively rated intensity of the taste (Grabenhorst and Rolls, 2008; Grabenhorst et al., 2008). The anterior insular site may be the primary taste cortex (Pritchard et al., 1986; Yaxley et al., 1990; de Araujo et al., 2003a; de Araujo and Rolls, 2004; Rolls, 2008a), and the mid-insular site a region activated by other oral including somatosensory and fat texture inputs from the oral cavity (de Araujo et al., 2003b; de Araujo and Rolls, 2004) and perhaps by taste *per se* (Small et al., 2003) in that the activations there were correlated with the trial-by-trial subjective ratings of the taste intensity made during the scanning (Grabenhorst and Rolls, 2008). In the analyses described here, such somatosensory inputs could contribute to the attention-dependent correlations found between the mid insula and other areas.

The interpretation of this functional connectivity revealed with PPI (Friston et al., 1997) is that the prefrontal cortex and orbitofrontal/pregenual cingulate areas covary in their activations more strongly when attention is directed to pleasantness than to intensity. In this study, the implication is that when the activity in the orbitofrontal and pregenual cingulate areas is high, as it is on trials when attention is paid to pleasantness relative to trials when attention is paid to intensity, then activations in this prefrontal cortex region are also high. A large source of this variation which gives rise to the PPI effect is thus the difference in the activations on different trial types which can be captured by the correlation arising from the difference in the mean activations of both sites (orbitofrontal/pregenual cingulate cortices and prefrontal cortex) on each of the two trial types (see further O'Reilly et al., 2012). However, in addition to this source of variation, it could be that when two areas are functionally interacting strongly, there may be an additional contribution to the connectivity term produced by the trial-by-trial variation within a type of trial. For example, on trials on which pleasantness is the subject of attention, then any small variation on a particular trial in the prefrontal cortex would be expected to be reflected in the activations in the orbitofrontal/pregenual cingulate cortex. This effect would arise because when both areas are active, the neurons in each area may be operating on a relatively linear part of their activation function, producing strong coupling, whereas when one or both areas are relatively inactive, with only spontaneous firing, then the neurons may be subject to some effects produced by being close to the firing threshold, such that small changes in input may produce a smaller than linear effect on the output. This trial-by-trial variation corresponds in information theoretic analysis of neuronal covariation to a “noise” effect as compared to a “signal” effect (Oram et al., 1998; Rolls et al., 2003a; Rolls, 2008b; Rolls and Treves, 2011).

### Granger causality used to investigate the source of the top-down biasing of affective processing

Correlations between signals, including signals at the neuronal or at the functional neuroimaging level, do not reveal the direction of the possible influence of one signal on the other. PPI analysis is based on correlations. Understanding how one brain area may influence another, for example by providing it with inputs, or by top-down modulation, is fundamental to understanding how the brain functions (Mechelli et al., 2004; Bar, 2007; Bressler and Menon, 2010). Hence, inferring causal influences from time series data has been attracting intensive interest. Recently, Granger causality has become increasingly popular due to its easy implementation and many successful applications to econometrics, neuroscience, etc., and in particular, the study of brain function (Ding et al., 2006; Bressler et al., 2008; Deshpande et al., 2010; Hwang et al., 2010; Schippers et al., 2010; Bressler and Seth, 2011; Jiao et al., 2011; Luo et al., 2011). The application of Granger causality analysis to BOLD fMRI signals which are inherently slow has been discussed elsewhere (David et al., 2008; Deshpande et al., 2010; Schippers and Keysers, 2011; Valdes-Sosa et al., 2011; Stephan and Roebroeck, 2012; Luo et al., in revision).

Granger causality is based on precedence and predictability. Originally proposed by Wiener (1956) and further formalized by Granger (1969), it states that given two times series *x* and *y*, if the inclusion of the past history of *y* helps to predict the future states of *x* in some plausible statistical sense, then *y* is a cause of *x* in the Granger sense. In spite of the wide acceptance of this definition, classical Granger causality is not tailored to measure the effects of interactions between time series *x* and *y* on the causal influences, and cannot measure systematically the effects of the past history of *x* on *x* (Ge et al., 2012). A componential form of Granger causality analysis has recently been introduced which has advantages over classical Granger analysis (Ge et al., 2012). Componential Granger causality measures the effect of *y* on *x*, but allows interaction effects between *y* and *x* to be measured (Ge et al., 2012). In addition, the terms in componential Granger causality sum to 1, allowing causal effects to be directly compared between systems.

**Figure 5:**
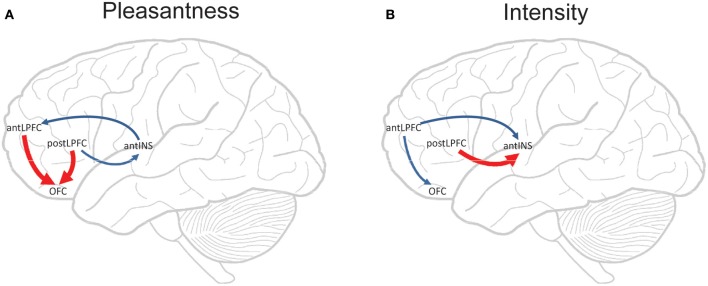
**Componential Granger causality analysis of top-down effects on taste processing from different lateral prefrontal cortex areas during attention to either the pleasantness (A) or to the intensity (B) of a taste.** Significant causal influences from *t*-tests with a Bonferroni correction are marked by blue arrows (i.e., cross-componential Granger causality is greater than 0). Red arrows indicate where significant top-down effects exist in addition to significant causal influences (i.e., a significant cross-componential Granger causality that is different in the two directions). The areas are anterior (mean *y* ≈ 50) and posterior (mean *y* ≈ 37) lateral prefrontal cortex (antLPFC, postLPFC); orbitofrontal cortex secondary cortical taste area (OFC); and anterior insular cortex primary cortical taste area (antINS). [Reproduced with permission from Ge et al. (2012)].

We showed using componential Granger causality analysis applied to an fMRI investigation that there is a top-down attentional effect from the anterior dorsolateral prefrontal cortex to the OFC when attention is paid to the pleasantness of a taste, and that this effect depends on the activity in the OFC as shown by the interaction term (Ge et al., 2012). Correspondingly there is a top-down attentional effect from the posterior dorsolateral prefrontal cortex to the insular primary taste cortex when attention is paid to the intensity of a taste, and this effect depends on the activity of the insular primary taste cortex as shown by the interaction term. The prefrontal cortex sites are those identified by the PPI analysis (Grabenhorst and Rolls, 2010) and the effects are shown schematically in Figure 5. Componential Granger causality thus not only can reveal the directionality of effects between areas (and these can be bidirectional), but also allows the mechanisms to be understood in terms of whether the causal influence of one system on another depends on the state of the system being causally influenced. Componential Granger causality measures the full effects of second order statistics by including variance and covariance effects between each time series, thus allowing interaction effects to be measured, and also provides a systematic framework within which to measure the effects of cross, self, and noise contributions to causality (Ge et al., 2012). The findings reveal some of the mechanisms involved in a biased activation theory of selective attention.

## A top-down biased activation theory of attentional and cognitive modulation

**Figure 6:**
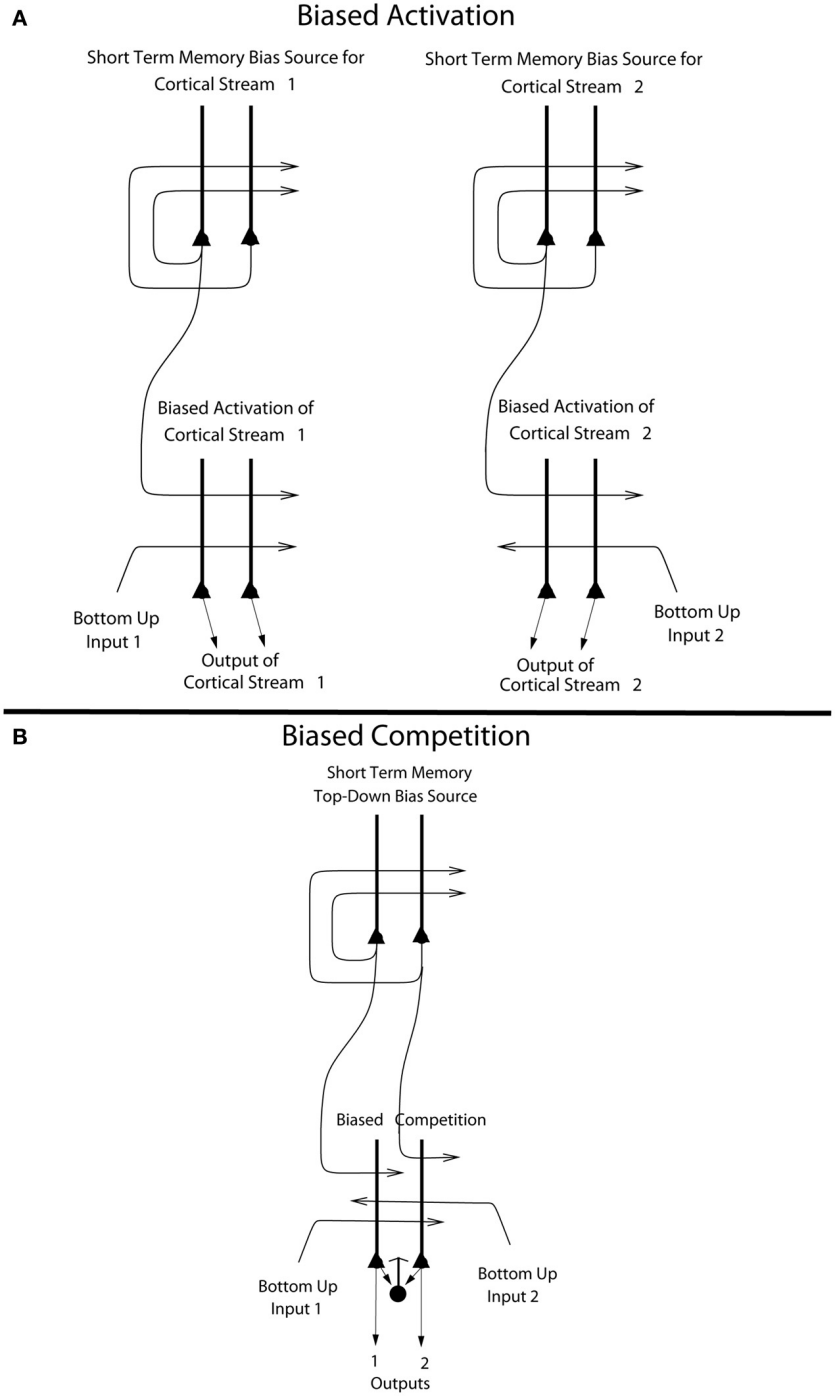
**(A)** Biased activation. The short-term memory systems that provide the source of the top-down activations may be separate (as shown), or could be a single network with different attractor states for the different selective attention conditions. The top-down short-term memory systems hold what is being paid attention to active by continuing firing in an attractor state, and bias separately either cortical processing system 1, or cortical processing system 2. This weak top-down bias interacts with the bottom up input to the cortical stream and produces an increase of activity that can be supralinear (Deco and Rolls, 2005b). Thus the selective activation of separate cortical processing streams can occur. In the example, stream 1 might process the affective value of a stimulus, and stream 2 might process the intensity and physical properties of the stimulus. The outputs of these separate processing streams then must enter a competition system, which could be for example a cortical attractor decision-making network that makes choices between the two streams, with the choice biased by the activations in the separate streams (see text). **(B)** Biased competition. There is usually a single attractor network that can enter different attractor states to provide the source of the top-down bias (as shown). If it is a single network, there can be competition within the short-term memory attractor states, implemented through the local GABA inhibitory neurons. The top-down continuing firing of one of the attractor states then biases in a top-down process some of the neurons in a cortical area to respond more to one than the other of the bottom-up inputs, with competition implemented through the GABA inhibitory neurons (symbolized by a filled circle) which make feedback inhibitory connections onto the pyramidal cells (symbolized by a triangle) in the cortical area. The thick vertical lines above the pyramidal cells are the dendrites. The axons are shown with thin lines and the excitatory connections by arrow heads.

The way that we think of top-down biased competition as operating normally in for example visual selective attention (Desimone and Duncan, 1995) is that within an area, e.g., a cortical region, some neurons receive a weak top-down input that increases their response to the bottom-up stimuli (Desimone and Duncan, 1995), potentially supralinearly if the bottom-up stimuli are weak (Rolls and Deco, 2002; Deco and Rolls, 2005a; Rolls, 2008b). The enhanced firing of the biased neurons then, via the local inhibitory neurons, inhibits the other neurons in the local area from responding to the bottom-up stimuli. This is a local mechanism, in that the inhibition in the neocortex is primarily local, being implemented by cortical inhibitory neurons that typically have inputs and outputs over no more than a few mm (Rolls and Deco, 2002; Douglas et al., 2004; Rolls, 2008b). This model of biased competition is illustrated in Figure 6B.

This locally implemented biased competition situation may not apply in the present case, where we have facilitation of processing in a whole cortical area (e.g., OFC, or pregenual cingulate cortex) or even cortical processing stream (e.g., the linked orbitofrontal and pregenual cingulate cortex) in which any taste neurons may reflect pleasantness and not intensity. So the attentional effect might more accurately be described in this case as biased activation, without local competition being part of the effect. This *biased activation theory and model of attention*, illustrated in Figure 6A, is a rather different way to implement attention in the brain than biased competition, and each mechanism may apply in different cases, or both mechanisms in some cases.

The *biased activation theory of top-down attentional and cognitive control* is as follows, and is illustrated in Figure 6A. There are short-term memory systems implemented as cortical attractor networks with recurrent collateral connections to maintain neuronal activity (Rolls, 2008b) that provide the source of the top-down activation. The short-term memory systems may be separate (as shown in Figure 6A), or could be a single network with different attractor states for the different selective attention conditions. The top-down short-term memory systems hold what is being paid attention to active by continuing firing in an attractor state, and bias separately either cortical processing system 1, or cortical processing system 2. This weak top-down bias interacts with the bottom-up input to the cortical stream and produces an increase of activity that can be supralinear (Deco and Rolls, 2005b; Rolls, 2008b). Thus the selective activation of separate cortical processing streams can occur. In the example, stream 1 might process the affective value of a stimulus, and stream 2 might process the intensity and physical properties of the stimulus.

**Figure 7:**
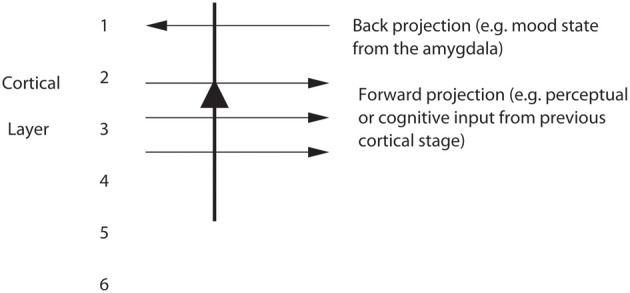
**Pyramidal cells in, for example, layers 2 and 3 of the temporal lobe association cortex receive forward inputs from preceding cortical stages of processing, and also backprojections from the amygdala.** It is suggested that the backprojections from the amygdala make modifiable synapses on the apical dendrites of cortical pyramidal cells during learning when amygdala neurons are active in relation to a mood state; and that the backprojections from the amygdala via these modified synapses allow mood state to influence later cognitive processing, for example by facilitating some perceptual representations.

The top-down bias needs to be weak relative to the bottom-up input, for the top-down bias must not dominate the system, otherwise bottom-up inputs, essential for perception and survival, would be over-ridden. Under such conditions, top-down attentional and cognitive effects will be largest when the bottom-up inputs are not too strong or are ambiguous, and that has been shown to be the case in realistic simulations with integrate-and-fire neurons (Deco and Rolls, 2005b; Rolls, 2008b). The weakness of the top-down biasing input is included as a part of brain design, for the top-down inputs are effectively backprojections from higher cortical areas, and these end on the apical dendrites of cortical pyramidal cells, and so have weaker effects than the bottom up inputs, which make connections lower down the dendrite toward the cell body (Rolls, 2008b) (see Figures 6, 7). I suggest here that the correct connections could be set up in such a system by the following associative (Hebbian) synaptic learning process. The top-down backprojection synapses would increase in strength when there is activity in a population of short-term memory neurons that by their firing hold attention in one direction (e.g., the short-term memory system for cortical stream 1 shown in Figure 6A), and simultaneously there is activity in the neurons that receive the top-down inputs (e.g., in cortical stream 1 shown in Figure 6A).

The outputs of the separate processing streams showing biased activation (Figure 6A) may need to be compared later to lead to a single behavior. One way in which this comparison could take place is by both outputs entering a single network cortical attractor model of decision-making, in which positive feedback implemented by the excitatory recurrent collateral connections leads through non-linear dynamics to a single winner, which is ensured by competition between the different possible attractor states produced through inhibitory neurons (Wang, 2002, 2008; Deco and Rolls, 2006; Rolls and Deco, 2010; Deco et al., 2012). A second way in which the competition could be implemented is by that usually conceptualized as important in biased competition (Desimone and Duncan, 1995; Rolls and Deco, 2002; Deco and Rolls, 2005a,b), in which a feedforward competitive network using inhibition through local inhibitory neurons provides a way for a weak top-down signal to bias the output especially if the bottom-up inputs are weak (Rolls and Deco, 2002; Deco and Rolls, 2005b; Rolls, 2008b), and this implementation is what is shown at the bottom of Figure 6B. A third way in which the biased activation reflected in the output of the streams shown in Figure 6A could be taken into account is by a mechanism such as that in the basal ganglia, where in the striatum the different excitatory inputs activate GABA (gamma-amino-butyric acid) neurons, which then directly inhibit each other to make the selection (Rolls, 2005, 2008b).

The difference between biased competition and biased activation may be especially important in the context of functional neuroimaging, for biased activation, in which processing in whole cortical areas is facilitated by selective attention, can be revealed by functional neuroimaging, which operates at relatively low spatial resolution, in the order of mm. In contrast, biased competition may selectively facilitate some pyramidal neurons within a local cortical area which then through the local GABA inhibitory neurons compete with the other pyramidal neurons in the area receiving bottom-up input. In this situation, in which some but not other neurons within a cortical area are showing enhanced firing, functional neuroimaging may not be able to show which local population of pyramidal cells is winning the competition due to the top-down bias. The evidence presented by Grabenhorst and Rolls (2010) is that not only the processing streams, but also even the short-term memory systems in the prefrontal cortex that provide the top-down source of the biased activation, are physically separate, as illustrated in Figure 5A.

A possibility arising from this model is that some competition may occur somewhere in the attentional system before the output stage, and one possible area is within the prefrontal cortex, where it is a possibility that the attractors that implement the short-term memory for attention to pleasantness (at Y ≈ 50) may inhibit the attractors that implement the short-term memory for attention to intensity (at Y ≈ 37), which could occur if there is some physical overlap between their zones of activation, even if the peaks are well separated. Some evidence for this possibility was found (Grabenhorst and Rolls, 2010), in that the correlation between the % BOLD activations in these two prefrontal cortex regions was *r* = −0.72 (*p* = 0.0034) on the pleasantness trials; and *r* = −0.8 (*p* < 0.001) on the intensity trials. In a biased competition model (Figure 6B) we would normally think of the short-term memory attractors that provide the source of the bias as being within the same single attractor network, so that there would be competition between the two attractor states through the local inhibitory interneurons. In the biased activation model (Figure 6A), it is an open issue about whether the attractors that provide the source of the top-down bias are in the same single network, or are physically separate making interactions between the attractor states difficult through the short-range cortical inhibitory neurons. The findings just described indicate that in the case of top-down control of affective vs. intensity processing of taste stimuli, although the two attractors are somewhat apart in the prefrontal cortex, there is some functional inhibitory interaction between them.

The principle of biased activation providing a mechanism for selective attention probably extends beyond processing in the affective vs. sensory coding cortical streams. It may provide the mechanism also for effects in for example the dorsal vs. the ventral visual system, in which attention to the motion of a moving object may enhance processing in the dorsal stream, and attention to the identity of the moving object may enhance processing in the ventral visual stream (Brown, 2009). Similar biased activation may contribute to the different localization in the prefrontal cortex of systems involved in “what” vs. “where” working memory (Deco et al., 2004; Rottschy et al., 2012). Biased activation as a mechanism for top-down selective attention may be widespread in the brain, and may be engaged when there is segregated processing of different attributes of stimuli (Grabenhorst and Rolls, 2010).

## A neurophysiological mechanism for top-down attention

We have developed an integrate-and-fire neuronal model of how top-down attentional effects operate at the neuronal level (Deco and Rolls, 2005b). The model has neurons with the membrane potential driven by the dynamically modeled synaptic currents (Brunel and Wang, 2001; Rolls, 2008b; Rolls and Deco, 2010), and allows biophysical properties of the ion channels affected by synapses, and of the membrane dynamics, to be incorporated, and shows how the non-linear interactions between bottom-up effects (produced for example by altering stimulus contrast) and top-down attentional effects can account for neurophysiological results in areas MT and V4 l (Deco and Rolls, 2005b). The model and simulations show that attention has its major modulatory effect at intermediate levels of bottom-up input, and that the effect of attention disappears at high levels of contrast of the competing stimulus.

The model assumes no kind of multiplicative attentional effects on the gain of neuronal responses. Instead, in the model, both top-down attention and bottom-up input information (contrast) are implemented in the same way, via additive synaptic effects in the postsynaptic neurons. There is of course a non-linearity in the effective activation function of the integrate-and-fire neurons, and this is what we identify as the source of the apparently multiplicative (Martinez-Trujillo and Treue, 2002) effects of top-down attentional biases on bottom-up inputs. The relevant part of the effective activation function of the neurons (the relation between the firing and the injected excitatory currents) is the threshold non-linearity, and the first steeply rising part of the activation function, where just above threshold the firing increases markedly with small increases in synaptic inputs (Brunel and Wang, 2001). Attention was therefore interpreted as a phenomenon that results from purely additive synaptic effects, non-linear effects in the neurons, and cooperation-competition dynamics in the network, which together yield a variety of modulatory effects, including effects that appear (Martinez-Trujillo and Treue, 2002) to be multiplicative. In addition, we were able to show that the non-linearity of the NMDA receptors may facilitate non-linear attentional effects, but is not necessary for them. This was shown by disabling the voltage-dependent non-linearity of the NMDA receptors in the simulations (Deco and Rolls, 2005b).

## Effects of emotion on cognitive processing

Emotional states can influence memory (McIntyre et al., 2012) and perception (Pessoa, 2010). A brain system where effects of emotional state and mood on storage and recall could be instantiated is in the backprojection system from structures important in emotion such as the amygdala and OFC to parts of the cerebral cortex important in the representation of objects, such as the inferior temporal visual cortex, and more generally, to parts of the cerebral cortex involved in storing memories. It is suggested (Rolls, 1989, 2008b; Treves and Rolls, 1994) that co-activity between forward inputs and backprojecting inputs to strongly activated cortical pyramidal cells would lead to both sets of synapses being modified (see Figure 7). This could result in facilitation or recall of cortical representations (for example of particular faces) that had become associated with emotional states, represented by activity in the amygdala).

**Figure 8:**
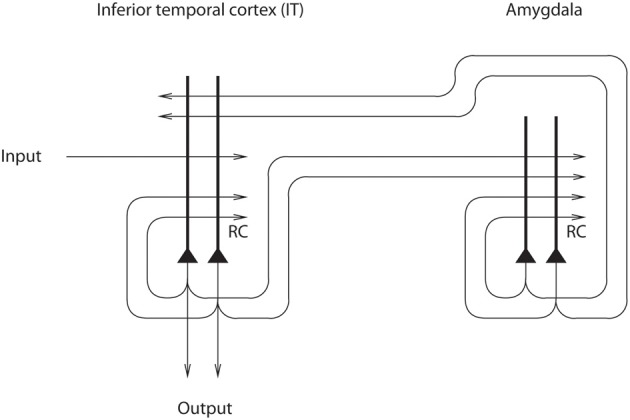
**Architecture used to investigate how mood can affect perception and memory.** The IT module represents brain areas such as the inferior temporal cortex involved in perception and hippocampus-related cortical areas that have forward connections to regions such as the amygdala and orbitofrontal cortex involved in mood and emotion (after Rolls and Stringer, 2001).

A theory of how the effects of mood on memory and perception could be implemented in the brain has been developed (Rolls, 1989, 1999, 2005) and tested (Rolls and Stringer, 2001). The architecture, shown in Figure 8, uses the massive backprojections from parts of the brain where mood is represented, such as the OFC and amygdala, to the cortical areas such as the inferior temporal visual cortex and hippocampus-related areas (labeled IT in Figure 8) that project into these mood-representing areas (Amaral et al., 1992). The model uses an attractor network (see Rolls, 2008b Appendix 2) in the mood module (labeled amygdala in Figure 8), which helps the mood to be an enduring state, and also an attractor in the inferior temporal visual cortex (IT) (or any other cortical area that receives backprojections from the amygdala or OFC). The system is treated as a system of coupled attractors (Rolls, 2008b), but with an odd twist: many different perceptual states are associated with any one mood state. Overall, there is a large number of perceptual/memory states, and only a few mood states, so that there is a many-to-one relation between perceptual/memory states and the associated mood states. The network displays the properties that one would expect [provided that the coupling parameters *g* for the synaptic strengths between the attractors are weak (Rolls, 2008b)]. These include the ability of a perceptual input to trigger a mood state in the “amygdala” module if there is not an existing mood, but greater difficulty to induce a new mood if there is already a strong mood attractor present; and the ability of the mood to affect via the backprojections which memories or perceptual states are triggered (Rolls and Stringer, 2001).

Another interesting finding was that the forward connections to the mood module from the memory module must be relatively strong, if new inputs to the memory module are to alter the firing in the mood module by overcoming an existing mood state being kept active by the recurrent collateral connections (Rolls and Stringer, 2001). These results are consistent with the general effects needed for forward and backward projections in the brain, namely that forward projections must be relatively strong in order to produce new firing in a module when a new (forward) input is received, and backward projections must be relatively weak, if they are to mildly implement “top-down” constraints without dominating the activity of earlier modules (Renart et al., 1999a,b, 2001; Rolls, 2008b). Consistent with this, forward projections terminate on cortical neurons closer to the cell body (where they can have a stronger influence) than backprojections (which typically terminate on the distal extremities of the apical dendrite of cortical neurons, in layer 1, the top layer of the cortex (Rolls, 2008b).

An interesting property that was revealed by the model is that because of the many-to-few mapping of perceptual to mood states, an effect of a mood was that it tended to make all the perceptual or memory states associated with a particular mood more similar then they would otherwise have been (Rolls and Stringer, 2001). The implication is that the coupling parameter *g* for the backprojections must be quite weak, as otherwise interference increases in the perceptual/memory module.

In summary, emotional states may affect whether or how strongly memories are stored using the basal forebrain memory strobe (Rolls, 2005); be stored as part of many memories in for example the hippocampus (Rolls, 2005); and may influence both the recall of such memories, and the operation of cognitive processing, using backprojections in the way described in the preceding paragraphs. In turn, cognitive inputs can influence affective states, as described earlier in this paper.

## Conclusions

We have thus seen that cognition can influence emotion by biasing neural activity in the first cortical region in which the reward value and subjective pleasantness of stimuli is made explicit in the representation, the OFC. The same effect occurs in a second cortical tier for emotion, the ACC. Similar effects are found for selective attention, to for example the pleasantness vs. the intensity of stimuli, which modulates representations of reward value and affect in the orbitofrontal and anterior cingulate cortices. The mechanisms for the effects of cognition and attention on emotion are top-down biased competition and top-down biased activation. Affective and mood states can in turn influence memory and perception, by backprojected biasing influences.

Emotion-related decision systems operate to choose between gene-specified rewards such as taste, touch, and beauty. Reasoning processes capable of planning ahead with multiple steps held in working memory in the explicit system can allow the gene-specified rewards not to be selected, or to be deferred (Rolls, 2014). The decisions between the selfish-gene-specified rewards, and the explicitly, cognitively, calculated rewards that are in the interests of the individual, the phenotype, may be influenced by the stochastic, noisy, dynamics of decision-making systems in the brain (Rolls and Deco, 2010; Rolls, 2013c).

### Conflict of interest statement

The author declares that the research was conducted in the absence of any commercial or financial relationships that could be construed as a potential conflict of interest.

## References

[B1] Amaral D. G. Price J. L. Pitkanen A. Carmichael S. T. (1992). Anatomical organization of the primate amygdaloid complex, in The Amygdala, ed Aggleton J. P. (New York, NY: Wiley-Liss), 1–66

[B2] Bar M. (2007). The proactive brain: using analogies and associations to generate predictions. Trends Cogn. Sci. 11, 280–289 10.1016/j.tics.2007.05.005 17548232

[B3] Beck D. M. Kastner S. (2009). Top-down and bottom-up mechanisms in biasing competition in the human brain. Vision Res. 49, 1154–1165 10.1016/j.visres.2008.07.012 18694779 PMC2740806

[B4] Bengtsson S. L. Haynes J. D. Sakai K. Buckley M. J. Passingham R. E. (2009). The representation of abstract task rules in the human prefrontal cortex. Cereb. Cortex 19, 1929–1936 10.1093/cercor/bhn222 19047573 PMC2705703

[B5] Bressler S. L. Menon V. (2010). Large-scale brain networks in cognition: emerging methods and principles. Trends Cogn. Sci. 14, 277–290 10.1016/j.tics.2010.04.004 20493761

[B6] Bressler S. L. Seth A. K. (2011). Wiener-Granger causality: a well established methodology. Neuroimage 58, 323–329 10.1016/j.neuroimage.2010.02.059 20202481

[B7] Bressler S. L. Tang W. Sylvester C. M. Shulman G. L. Corbetta M. (2008). Top-down control of human visual cortex by frontal and parietal cortex in anticipatory visual spatial attention. J. Neurosci. 28, 10056–10061 10.1523/JNEUROSCI.1776-08.2008 18829963 PMC2583122

[B8] Brown J. M. (2009). Visual streams and shifting attention. Prog. Brain Res. 176, 47–63 10.1016/S0079-6123(09)17604-5 19733749

[B9] Brunel N. Wang X. J. (2001). Effects of neuromodulation in a cortical network model of object working memory dominated by recurrent inhibition. J. Comput. Neurosci. 11, 63–85 11524578 10.1023/a:1011204814320

[B10] David O. Guillemain I. Saillet S. Reyt S. Deransart C. Segebarth C. (2008). Identifying neural drivers with functional MRI: an electrophysiological validation. PLoS Biol. 6:e315 10.1371/journal.pbio.0060315 19108604 PMC2605917

[B11] de Araujo I. E. T. Kringelbach M. L. Rolls E. T. Hobden P. (2003a). The representation of umami taste in the human brain. J. Neurophysiol. 90, 313–319 10.1152/jn.00669.2002 12843312

[B12] de Araujo I. E. T. Kringelbach M. L. Rolls E. T. McGlone F. (2003b). Human cortical responses to water in the mouth, and the effects of thirst. J. Neurophysiol. 90, 1865–1876 10.1152/jn.00297.2003 12773496

[B13] de Araujo I. E. T. Rolls E. T. (2004). The representation in the human brain of food texture and oral fat. J. Neurosci. 24, 3086–3093 10.1523/JNEUROSCI.0130-04.2004 15044548 PMC6729847

[B14] de Araujo I. E. T. Rolls E. T. Velazco M. I. Margot C. Cayeux I. (2005). Cognitive modulation of olfactory processing. Neuron 46, 671–679 10.1016/j.neuron.2005.04.021 15944134

[B15] Deco G. Rolls E. T. (2005a). Attention, short-term memory, and action selection: a unifying theory. Prog. Neurobiol. 76, 236–256 10.1016/j.pneurobio.2005.08.004 16257103

[B16] Deco G. Rolls E. T. (2005b). Neurodynamics of biased competition and co-operation for attention: a model with spiking neurons. J. Neurophysiol. 94, 295–313 10.1152/jn.01095.2004 15703227

[B17] Deco G. Rolls E. T. (2006). Decision-making and Weber's law: a neurophysiological model. Eur. J. Neurosci. 24, 901–916 10.1111/j.1460-9568.2006.04940.x 16930418

[B18] Deco G. Rolls E. T. Horwitz B. (2004). “What” and “where” in visual working memory: a computational neurodynamical perspective for integrating fMRI and single-neuron data. J. Cogn. Neurosci. 16, 683–701 10.1162/089892904323057380 15165356

[B18a] Deco G. Rolls E. T. Albantakis L. Romo R. (2012). Brain mechanisms for perceptual and reward-related decision-making. Prog. Neurobiol. [Epub ahead of print]. 10.1016/j.pneurobio.2012.01.010 22326926

[B19] Deshpande G. Hu X. Lacey S. Stilla R. Sathian K. (2010). Object familiarity modulates effective connectivity during haptic shape perception. Neuroimage 49, 1991–2000 10.1016/j.neuroimage.2009.08.052 19732841 PMC3073838

[B20] Desimone R. Duncan J. (1995). Neural mechanisms of selective visual attention. Annu. Rev. Neurosci. 18, 193–222 10.1146/annurev.ne.18.030195.001205 7605061

[B21] Ding M. Chen Y. Bressler S. L. (2006). Granger causality: basic theory and application to neuroscience, in Handbook of Time Series Analysis, eds Schelter B. Winterhalder M. Timmer J. (Weinheim: Wiley), 437–460

[B22] Douglas R. J. Markram H. Martin K. A. C. (2004). Neocortex, in The Synaptic Organization of the Brain, 5th Edn, ed Shepherd G. M. (Oxford: Oxford University Press), 499–558

[B23] Etkin A. Egner T. Kalisch R. (2011). Emotional processing in anterior cingulate and medial prefrontal cortex. Trends Cogn. Sci. 15, 85–93 10.1016/j.tics.2010.11.004 21167765 PMC3035157

[B24] Friston K. J. Buechel C. Fink G. R. Morris J. Rolls E. T. Dolan R. J. (1997). Psychophysiological and modulatory interactions in neuroimaging. Neuroimage 6, 218–229 10.1006/nimg.1997.0291 9344826

[B25] Ge T. Feng J. Grabenhorst F. Rolls E. T. (2012). Componential Granger causality, and its application to identifying the source and mechanisms of the top-down biased activation that controls attention to affective vs sensory processing. Neuroimage 59, 1846–1858 10.1016/j.neuroimage.2011.08.047 21888980

[B26] Grabenhorst F. Rolls E. T. (2008). Selective attention to affective value alters how the brain processes taste stimuli. Eur. J. Neurosci. 27, 723–729 10.1111/j.1460-9568.2008.06033.x 18279324

[B27] Grabenhorst F. Rolls E. T. (2010). Attentional modulation of affective vs sensory processing: functional connectivity and a top-down biased activation theory of selective attention. J. Neurophysiol. 104, 1649–1660 10.1152/jn.00352.2010 20631210

[B28] Grabenhorst F. Rolls E. T. (2011). Value, pleasure, and choice in the ventral prefrontal cortex. Trends Cogn. Sci. 15, 56–67 10.1016/j.tics.2010.12.004 21216655

[B29] Grabenhorst F. Rolls E. T. Bilderbeck A. (2008). How cognition modulates affective responses to taste and flavor: top down influences on the orbitofrontal and pregenual cingulate cortices. Cereb. Cortex 18, 1549–1559 10.1093/cercor/bhm185 18056086

[B30] Granger C. W. J. (1969). Investigating causal relations by econometric models and cross-spectral methods. Econometrica 37, 414–438

[B31] Hwang K. Velanova K. Luna B. (2010). Strengthening of top-down frontal cognitive control networks underlying the development of inhibitory control: a functional magnetic resonance imaging effective connectivity study. J. Neurosci. 30, 15535–15545 10.1523/JNEUROSCI.2825-10.2010 21084608 PMC2995693

[B32] Jiao Q. Lu G. Zhang Z. Zhong Y. Wang Z. Guo Y. (2011). Granger causal influence predicts BOLD activity levels in the default mode network. Hum. Brain Mapp. 32, 154–161 10.1002/hbm.21065 21157880 PMC6870036

[B33] Johansen-Berg H. Lloyd D. M. (2000). The physiology and psychology of selective attention to touch. Front. Biosci. 5, D894–D904 11056079 10.2741/A558

[B34] Kobayashi S. (2012). Organization of neural systems for aversive information processing: pain, error, and punishment. Front. Neurosci. 6:136 10.3389/fnins.2012.00136 23049496 PMC3448295

[B35] Kouneiher F. Charron S. Koechlin E. (2009). Motivation and cognitive control in the human prefrontal cortex. Nat. Neurosci. 12, 939–945 10.1038/nn.2321 19503087

[B36] Kringelbach M. L. O'Doherty J. Rolls E. T. Andrews C. (2003). Activation of the human orbitofrontal cortex to a liquid food stimulus is correlated with its subjective pleasantness. Cereb. Cortex 13, 1064–1071 10.1093/cercor/13.10.1064 12967923

[B37] Lindquist K. A. Wager T. D. Kober H. Bliss-Moreau E. Barrett L. F. (2012). The brain basis of emotion: a meta-analytic review. Behav. Brain Sci. 35, 121–143 10.1017/S0140525X11000446 22617651 PMC4329228

[B38] Luo Q. Ge T. Feng J. (2011). Granger causality with signal-dependent noise. Neuroimage 57, 1422–1429 10.1016/j.neuroimage.2011.05.054 21645623

[B40] Martinez-Trujillo J. Treue S. (2002). Attentional modulation strength in cortical area MT depends on stimulus contrast. Neuron 35, 365–370 10.1016/S0896-6273(02)00778-X 12160753

[B41] McCabe C. Rolls E. T. Bilderbeck A. McGlone F. (2008). Cognitive influences on the affective representation of touch and the sight of touch in the human brain. Soc. Cogn. Affect. Neurosci. 3, 97–108 10.1093/scan/nsn005 19015100 PMC2555465

[B42] McIntyre C. K. McGaugh J. L. Williams C. L. (2012). Interacting brain systems modulate memory consolidation. Neurosci. Biobehav. Rev. 36, 1750–1762 10.1016/j.neubiorev.2011.11.001 22085800 PMC3315607

[B43] Mechelli A. Price C. J. Friston K. J. Ishai A. (2004). Where bottom-up meets top-down: neuronal interactions during perception and imagery. Cereb. Cortex 14, 1256–1265 10.1093/cercor/bhh087 15192010

[B44] Ochsner K. N. Silvers J. A. Buhle J. T. (2012). Functional imaging studies of emotion regulation: a synthetic review and evolving model of the cognitive control of emotion. Ann. N.Y. Acad. Sci. 1251, E1–E24 10.1111/j.1749-6632.2012.06751.x 23025352 PMC4133790

[B45] O'Doherty J. Kringelbach M. L. Rolls E. T. Hornak J. Andrews C. (2001). Abstract reward and punishment representations in the human orbitofrontal cortex. Nat. Neurosci. 4, 95–102 10.1038/82959 11135651

[B46] Oram M. W. Foldiak P. Perrett D. I. Sengpiel F. (1998). The ‘Ideal Homunculus’: decoding neural population signals. Trends Neurosci. 21, 259–265 10.1016/S0166-2236(97)01216-2 9641539

[B47] O'Reilly J. X. Woolrich M. W. Behrens T. E. Smith S. M. Johansen-Berg H. (2012). Tools of the trade: psychophysiological interactions and functional connectivity. Soc. Cogn. Affect. Neurosci. 7, 604–609 10.1093/scan/nss055 22569188 PMC3375893

[B48] Passingham R. E. P. Wise S. P. (2012). The Neurobiology of the Prefrontal Cortex. Oxford: Oxford University Press

[B49] Pessoa L. (2009). How do emotion and motivation direct executive control? Trends Cogn. Sci. 13, 160–166 10.1016/j.tics.2009.01.006 19285913 PMC2773442

[B50] Pessoa L. (2010). Emergent processes in cognitive-emotional interactions. Dialogues Clin. Neurosci. 12, 433–448 21319489 10.31887/DCNS.2010.12.4/lpessoa PMC3117594

[B51] Price J. L. (2006). Connections of orbital cortex, in The Orbitofrontal Cortex, eds Zald D. H. Rauch S. L. (Oxford: Oxford University Press), 39–55

[B52] Pritchard T. C. Hamilton R. B. Morse J. R. Norgren R. (1986). Projections of thalamic gustatory and lingual areas in the monkey, *Macaca fascicularis*. J. Comp. Neurol. 244, 213–228 10.1002/cne.902440208 3950095

[B53] Renart A. Moreno R. de la Rocha J. Parga N. Rolls E. T. (2001). A model of the IT-PF network in object working memory which includes balanced persistent activity and tuned inhibition. Neurocomputing 38–40, 1525–1531

[B54] Renart A. Parga N. Rolls E. T. (1999a). Associative memory properties of multiple cortical modules. Network 10, 237–255 10496475

[B55] Renart A. Parga N. Rolls E. T. (1999b). Backprojections in the cerebral cortex: implications for memory storage. Neural Comput. 11, 1349–1388 10423499 10.1162/089976699300016278

[B56] Rolls E. T. (1989). Functions of neuronal networks in the hippocampus and neocortex in memory, in Neural Models of Plasticity: Experimental and Theoretical Approaches, eds Byrne J. H. Berry W. O. (San Diego, CA: Academic Press), 240–265

[B57] Rolls E. T. (1999). The Brain and Emotion. Oxford: Oxford University Press

[B58] Rolls E. T. (2005). Emotion Explained. Oxford: Oxford University Press

[B59] Rolls E. T. (2008a). Functions of the orbitofrontal and pregenual cingulate cortex in taste, olfaction, appetite and emotion. Acta Physiol. Hung. 95, 131–164 10.1556/APhysiol.95.2008.2.1 18642756

[B60] Rolls E. T. (2008b). Memory, Attention, and Decision-Making: A Unifying Computational Neuroscience Approach. Oxford: Oxford University Press

[B61] Rolls E. T. (2010). The affective and cognitive processing of touch, oral texture, and temperature in the brain. Neurosci. Biobehav. Rev. 34, 237–245 10.1016/j.neubiorev.2008.03.010 18468687

[B62] Rolls E. T. (2011a). Consciousness, decision-making, and neural computation, in Perception-Action Cycle: Models, Algorithms and Systems, eds Cutsuridis V. Hussain A. Taylor J. G. (Berlin: Springer), 287–333

[B63] Rolls E. T. (2011b). A neurobiological basis for affective feelings and aesthetics, in The Aesthetic Mind: Philosophy and Psychology, eds Schellekens E. Goldie P. (Oxford: Oxford University Press), 116–165

[B64] Rolls E. T. (2012). Taste, olfactory, and food texture reward processing in the brain and the control of appetite. Proc. Nutr. Soc. 71, 488–501 10.1017/S0029665112000821 22989943

[B65] Rolls E. T. (2013a). Central neural integration of taste, smell and other sensory modalities, in Handbook of Olfaction and Gustation: Modern Perspectives, 3rd Edn, ed Doty R. L. (New York, NY: Dekker). 10.1007/s00221-005-2376-9

[B66] Rolls E. T. (2013b). What are emotional states, and why do we have them? Emot. Rev. 5

[B67] Rolls E. T. (2013c). Willed action, free will, and the stochastic neurodynamics of decision-making. Front. Integr. Neurosci. 6:68 10.3389/fnint.2012.00068 22973205 PMC3435521

[B68] Rolls E. T. (2014). Emotion and Decision-Making Explained. Oxford: Oxford University Press (in press).

[B69] Rolls E. T. Deco G. (2002). Computational Neuroscience of Vision. Oxford: Oxford University Press

[B70] Rolls E. T. Deco G. (2010). The Noisy Brain: Stochastic Dynamics as a Principle of Brain Function. Oxford: Oxford University Press 10.1016/j.pneurobio.2009.01.006 19428958

[B71] Rolls E. T. Franco L. Aggelopoulos N. C. Reece S. (2003a). An information theoretic approach to the contributions of the firing rates and correlations between the firing of neurons. J. Neurophysiol. 89, 2810–2822 10.1152/jn.01070.2002 12611978

[B72] Rolls E. T. Kringelbach M. L. de Araujo I. E. T. (2003b). Different representations of pleasant and unpleasant odors in the human brain. Eur. J. Neurosci. 18, 695–703 10.1046/j.1460-9568.2003.02779.x 12911766

[B73] Rolls E. T. O'Doherty J. Kringelbach M. L. Francis S. Bowtell R. McGlone F. (2003c). Representations of pleasant and painful touch in the human orbitofrontal and cingulate cortices. Cereb. Cortex 13, 308–317 10.1093/cercor/13.3.308 12571120

[B74] Rolls E. T. Grabenhorst F. Margot C. da Silva M. A. Velazco M. I. (2008a). Selective attention to affective value alters how the brain processes olfactory stimuli. J. Cogn. Neurosci. 20, 1815–1826 10.1162/jocn.2008.20128 18370603

[B75] Rolls E. T. Grabenhorst F. Parris B. A. (2008b). Warm pleasant feelings in the brain. Neuroimage 41, 1504–1513 10.1016/j.neuroimage.2008.03.005 18468458

[B76] Rolls E. T. Stringer S. M. (2001). A model of the interaction between mood and memory. Netw. Comput. Neural Syst. 12, 111–129 11405424

[B76a] Rolls E. T. Treves A. (2011). The neuronal encoding of information in the brain. Prog. Neurobiol. 95, 448–490 10.1016/j.pneurobio.2011.08.002 21907758

[B77] Rossi A. F. Pessoa L. Desimone R. Ungerleider L. G. (2009). The prefrontal cortex and the executive control of attention. Exp. Brain. Res. 192, 489–497 10.1007/s00221-008-1642-z 19030851 PMC2752881

[B78] Rottschy C. Langner R. Dogan I. Reetz K. Laird A. R. Schulz J. B. (2012). Modelling neural correlates of working memory: a coordinate-based meta-analysis. Neuroimage 60, 830–846 10.1016/j.neuroimage.2011.11.050 22178808 PMC3288533

[B79] Sakai K. Passingham R. E. (2003). Prefrontal interactions reflect future task operations. Nat. Neurosci. 6, 75–81 10.1038/nn987 12469132

[B80] Sakai K. Passingham R. E. (2006). Prefrontal set activity predicts rule-specific neural processing during subsequent cognitive performance. J. Neurosci. 26, 1211–1218 10.1523/JNEUROSCI.3887-05.2006 16436608 PMC6674561

[B81] Schippers M. B. Keysers C. (2011). Mapping the flow of information within the putative mirror neuron system during gesture observation. Neuroimage 57, 37–44 10.1016/j.neuroimage.2011.02.018 21316466

[B82] Schippers M. B. Roebroeck A. Renken R. Nanetti L. Keysers C. (2010). Mapping the information flow from one brain to another during gestural communication. Proc. Natl. Acad. Sci. U.S.A. 107, 9388–9393 10.1073/pnas.1001791107 20439736 PMC2889063

[B83] Scott T. R. Small D. M. (2009). The role of the parabrachial nucleus in taste processing and feeding. Ann. N.Y. Acad. Sci. 1170, 372–377 10.1111/j.1749-6632.2009.03906.x 19686161

[B84] Shackman A. J. Salomons T. V. Slagter H. A. Fox A. S. Winter J. J. Davidson R. J. (2011). The integration of negative affect, pain and cognitive control in the cingulate cortex. Nat. Rev. Neurosci. 12, 154–167 10.1038/nrn2994 21331082 PMC3044650

[B85] Sheppes G. Scheibe S. Suri G. Radu P. Blechert J. Gross J. J. (2012). Emotion regulation choice: a conceptual framework and supporting evidence. J. Exp. Psychol. Gen. [Epub ahead of print]. 10.1037/a0030831 23163767

[B86] Small D. M. Gregory M. D. Mak Y. E. Gitelman D. Mesulam M. M. Parrish T. (2003). Dissociation of neural representation of intensity and affective valuation in human gustation. Neuron 39, 701–711 10.1016/S0896-6273(03)00467-7 12925283

[B87] Stephan K. E. Roebroeck A. (2012). A short history of causal modeling of fMRI data. Neuroimage 62, 856–863 10.1016/j.neuroimage.2012.01.034 22248576

[B88] Treves A. Rolls E. T. (1994). A computational analysis of the role of the hippocampus in memory. Hippocampus 4, 374–391 10.1002/hipo.450040319 7842058

[B89] Valdes-Sosa P. A. Roebroeck A. Daunizeau J. Friston K. (2011). Effective connectivity: influence, causality and biophysical modeling. Neuroimage 58, 339–361 10.1016/j.neuroimage.2011.03.058 21477655 PMC3167373

[B90] Veldhuizen M. G. Bender G. Constable R. T. Small D. M. (2007). Trying to detect taste in a tasteless solution: modulation of early gustatory cortex by attention to taste. Chem. Senses 32, 569–581 10.1093/chemse/bjm025 17495173

[B91] Wang X. J. (2002). Probabilistic decision making by slow reverberation in cortical circuits. Neuron 36, 955–968 10.1016/S0896-6273(02)01092-9 12467598

[B92] Wang X. J. (2008). Decision making in recurrent neuronal circuits. Neuron 60, 215–234 10.1016/j.neuron.2008.09.034 18957215 PMC2710297

[B93] Wiener N. (1956). The theory of prediction, Chapter 8, in Modern Mathematics for Engineers, ed Beckenbach E. (New York, NY: McGraw-Hill), 165–190

[B94] Wise S. P. (2008). Forward frontal fields: phylogeny and fundamental function. Trends Neurosci. 31, 599–608 10.1016/j.tins.2008.08.008 18835649 PMC2587508

[B95] Yaxley S. Rolls E. T. Sienkiewicz Z. J. (1990). Gustatory responses of single neurons in the insula of the macaque monkey. J. Neurophysiol. 63, 689–700 2341869 10.1152/jn.1990.63.4.689

